# Cool White Polymer Coatings based on Glass Bubbles for Buildings

**DOI:** 10.1038/s41598-020-63027-2

**Published:** 2020-04-20

**Authors:** Xiao Nie, Youngjae Yoo, Hasitha Hewakuruppu, Jonathan Sullivan, Anirudh Krishna, Jaeho Lee

**Affiliations:** 1 Department of Mechanical and Aerospace Engineering, University of California, Irvine, CA 92617 USA; 2 Advanced Materials Division, Korea Research Institute of Chemical Technology, Daejeon, 34114 Korea

**Keywords:** Energy science and technology, Optical materials and structures, Materials for energy and catalysis, Materials for optics

## Abstract

While most selective emitter materials are inadequate or inappropriate for building applications, here we present a techno-economically viable optical coating by integrating glass bubbles within a polymer film. A controlled glass bubble volume concentration from 0 to 70% leads to a selective solar reflectivity increase from 0.06 to 0.92 while the mid-infrared emissivity remains above 0.85. Outdoor measurements show the polymer coating on a concrete surface can provide a temperature reduction up to 25 °C during the day when conduction and convection are limited and a net cooling power greater than 78 W/m^2^ at a cost less than $0.005/W. The impact of polymer coating on common buildings is estimated as potential annual energy savings of 2–12 MJ/m^2^ and CO_2_ emission savings of 0.3–1.5 kg/m^2^. More savings are expected for higher surface-area-to-volume-ratio buildings, and the polymer coating is also expected to resolve cooling issues for old buildings with no air conditioning.

## Introduction

It is expected that more than 6 billion people will live in urban environments by 2050^1^, and the aggressive urban infrastructure growth to accommodate larger population may result in critical side effects in energy consumption, air quality, and even human health. In particular, the annual cooling or air conditioning energy demand is forecasted to exceed ten quintillion (10^19^) joules by 2040^2^. and the associated carbon emissions and air pollution may play a critical role in early death, which is linked to 6.5 million premature deaths across the world in 2015^3^. On the other hand, the lack of air conditioning in old European building led to thousands of deaths estimated during the record-breaking heat wave in the summer 2019^4^.

Resolving the interconnected energy, greenhouse gas emission, air quality, and human health problems will require highly innovative approaches to surface cooling and advanced materials for use in urban environments. As a potential solution, engineering building surfaces^5,6^ to passively control thermal radiation or the concept of radiative cooling has drawn much attention in the recent years^6,7^. While the incoming solar irradiation^8^ within the wavelength (λ) range of 0.3 μm to 2.5 μm heats up the surface, radiation from the object to the cold outer space through the atmosphere’s transmission window^9^ in the mid-infrared (mid-IR) wavelength range of 8 μm to 13 μm helps cool the surface. Optical coating materials for radiative cooling require a high solar reflectivity to minimize heating by the sun and a high mid-infrared (IR) emissivity to maximize thermal emission from the surface to the atmosphere and to the cold space. This novel method decreases the amount of energy required for the active cooling of large-area outdoor buildings which cannot avoid intensive exposure to the solar irradiation and need to maintain a controlled thermal environment. While some nanophotonic structures including silicon (Si) or silicon dioxide (SiO_2_)-based layered structures^10^ have demonstrated substantial radiative cooling, the processing requirements and related costs might be inappropriate for building applications. Other approaches could include using dielectric pigments-embedded paints^11–13^, but near-infrared (NIR) and ultraviolet (UV) absorption are not attractive. Polymer composites^14–16^ can also achieve significant radiative cooling, but the use of expensive metallic films and complicated process may limit large-scale applications. For example, the silver-coated polymer composite^14^ can cost $2.49/m^2^ or $0.027/W while providing 93 W/m^2^ cooling power. The use of simple process and inexpensive materials is necessary. Daytime radiative cooling capability has been demonstrated both experimentally and theoretically, using randomly packed SiO_2_ microspheres^17^, hierarchal coating designs^18,19^ and bubbles made of pure glass^20^ or organic polymer^21^, to enable more hierarchical control of the polymer coatings. For large-scale lightweight structures, glass bubbles can be considered a good substitute for solid glass microspheres considering to their low density and high interface-to-volume ratio.

Here we present low-cost and scalable polymer coatings by integrating a controlled volume concentration of glass bubbles within a polymer (polydimethylsiloxane, PDMS) matrix and show a significant radiative cooling capability for buildings. Our polymer coating achieves a radiative cooling performance that is comparable or better compared to previously reported designs that required costly materials or processes^10,14,15^. We then use a building energy analysis tool to estimate the impact of cool white polymer coatings on building energy savings, and associated cost savings and carbon dioxide (CO_2_) emission savings. We perform techno-economic evaluations in terms of cost per area and cost per cooling power for our polymer coating material, commercial white paints and the state-of-the-art polymer coating solution.

## Results

### Section 1. Preparation, characterization and density analysis

**Figure 1:**
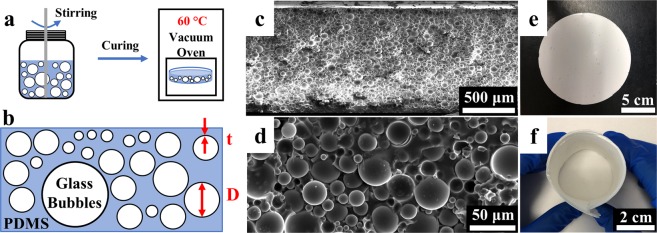
The fabrication and characterization of polymer coatings. (**a**) The schematic of the fabrication process of the polymer coatings; (**b**) The schematic of the polymer coating in which glass bubbles take up certain volume concentration inside PDMS matrix. The diameters (D) of glass bubbles are from 1 μm to 40 μm, with a mean value of 10 μm. The shell thicknesses (t) of glass bubbles are ranging from around 0.05 μm to 2 μm, and volume concentrations of glass bubbles inside the PDMS matrix are from 0 to 70% during our fabrication process; (**c**) The low-magnification and (**d**) high magnification cross-sectional SEM image of the as-prepared 70 vol% polymer coating; (**e**) Top-down view of the circular 70 vol% polymer coating sample with a dimeter of 5.5 inches; (**f**) The rolled 70 vol% polymer coating to demonstrate the flexibility.

We prepared polymer coatings (Fig. 1a) with a varying volume concentration (ϕ) of 0 to 70% (denoted as 0 to 70 vol%). The volume concentration (ϕ) of glass bubbles inside the polymer coating is defined as

1 $$\phi =\frac{{{\rm{V}}}_{{\rm{HGM}}}}{{{\rm{V}}}_{{\rm{PC}}}}$$

where V_HGM_ is the volume of glass bubbles, and V_PC_ is the volume of the polymer coating.

Figure 1b shows a cross-sectional schematic of the polymer coating, in which glass bubbles occupy certain ϕ, changing the film to an opaque appearance without sacrificing the robustness and lightweight attributes of pure PDMS. Figs. S1a to S1d shows the Scanning Electron Microscope (SEM) images and the corresponding size analysis of glass bubbles. The size measurements and analysis indicate that the diameters of glass bubbles (D) range from 1 μm to over 40 μm and center at ~8 μm. The mean value of the glass bubbles’ diameters is around 10 μm, which is supported by size analysis of multiple SEM images in different observation locations. From the SEM observation, the shell thicknesses of glass bubbles (t) are roughly from 0.05 μm to 2 μm. After being mixed with PDMS, the glass bubbles distribute from 1 μm to 50 μm for over 95% microspheres and the mean value is 12 μm (Fig. S1e), as studied by nano Computed Tomography (CT) which uses X-rays to detect the cross-sections from a three-dimensional polymer coating that are later used to recreate a virtual model (inset in Fig. S1e).

It is also observed that the glass bubble has very thin shell with a large air void inside, which leads to an extremely low mass density (0.460 g/cm^3^, or 460 kg/m^3^). As we prepare polymer coatings by integrating glass bubbles within uncured PDMS – which has a higher theoretical mass density (0.965 g/cm^3^, or 965 kg/m^3^) – it should be noted that the larger ϕ of glass bubbles in the polymer coating, the lower mass density the mixture possesses. We prepare polymer coatings with varying ϕ, ranging from 3 to 70%. As shown in Table S1, the measured mass density of the polymer coating drops from 905 kg/m^3^ to 681 kg/m^3^. Regarding the areal density, 3 vol% with a thickness of 330 μm results in 0.306 kg/m^2^, while 70 vol% with a thickness of 1300 μm leads to 0.885 kg/m^2^. The theoretical mass density (ρ) of the polymer coating in Table S1 can be obtained as a function of ϕ of glass bubbles by

2 $$\rho \left(\frac{kg}{{m}^{3}}\right)=\frac{(100-\phi )}{100}\ast 965+\frac{\phi }{100}\ast 460$$

After blending with uncured PDMS, glass bubbles are randomly distributed and take up a large area inside the polymer matrix, according to the cross-sectional SEM images of the 70 vol% polymer coating in Fig. 1c,d. Since the mixture shows a paint-like format, the final shape of the polymer coating depends on the shape of the substrate. This feature can be utilized to fabricate any arbitrary shape desired, and it indicates the possibility of fabricating a large-scale polymer coating that meets the massive needs for surface radiative thermal management when combined with advanced manufacturing techniques such as blade coating. We therefore achieve the 5.5 inch-diameter circular sample preparation, as indicated in Fig. 1e. Furthermore, the polymer coating can be twisted or rolled to meet any special practical needs; even with a 70 vol%, the sample demonstrates an excellent flexibility (Fig. 1f**)**. Mechanical properties can be very important for polymer coating applications with regards to reliability and stability. We observed that that the stiffness and the Young’s modulus of the polymer composites increase as the volume concentration of glass bubbles increases, but the stretchability and the flexibility decrease.

### Section 2. Optical properties of polymer coatings

**Figure 2:**
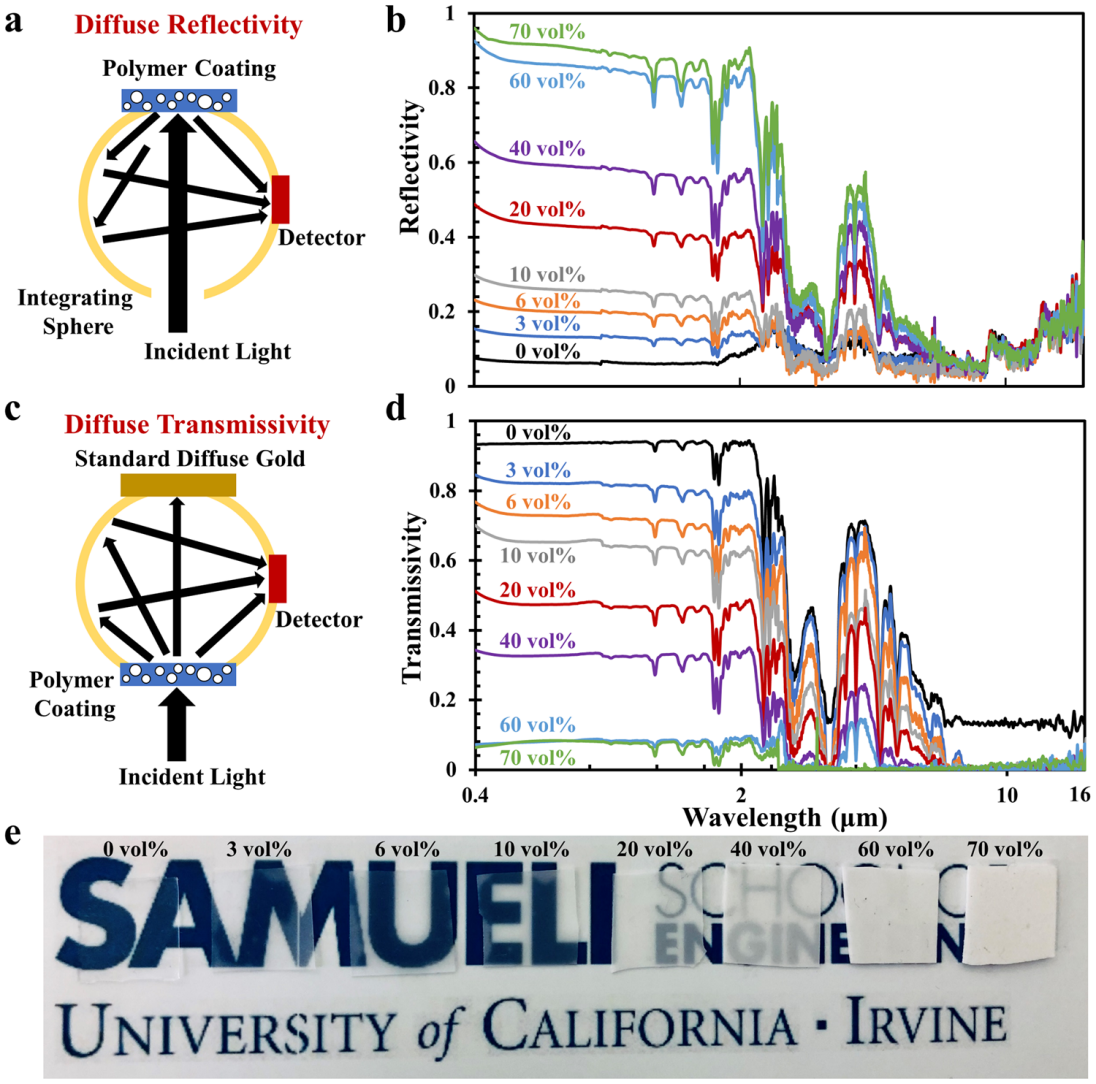
The measured diffuse reflectivity and transmissivity of the polymer coatings with varying ϕ in the wavelength range of 0.4 µm to 16 µm. (**a,b**) Diffuse reflectivity and (**c,d**) transmissivity of the polymer coatings with ϕ from 0 to 70% in the wavelength range of 0.4 µm to 16 µm. An integrating sphere is used to account for the scattered light from the full solid angle in the measurements. For (**a**) diffuse reflectivity, the polymer coating is placed on the reflection port of the integrating sphere in which all the backscattered light bounces off the integrating sphere surface several times until detected by the detector. For (**c**) diffuse transmissivity, the polymer coating is placed on the front port of the integrating sphere, allowing all the light transmitted through the polymer coating to be received by the detector; (**e**) Optical images of polymer coatings with increasing ϕ of glass bubbles from 0 vol% (left) to 70 vol% (right).

As the volume concentration (ϕ) of glass bubbles increases within a polymer matrix, the reflectivity increases selectively within the solar spectrum while remaining low in mid-IR wavelengths. We attribute the increasing solar reflectivity to increasing number of interfaces in the polymer coating and consequent increasing backscattering efficiency, which is confirmed by Ultraviolet-Visible-NIR (UV-VIS-NIR) spectroscopic measurement (λ, 0.4 μm to 2.5 μm). The mid-IR optical properties of polymer coatings with varying ϕ from 0 to 70% are characterized using a Fourier-transform infrared (FTIR) spectrometer in the mid-IR region (λ, 2.5 μm to 16 μm). We measured the diffuse reflectivity and transmissivity of polymer coatings with varying ϕ from 0 to 70% with integrating spheres which are used to account for the scattered light from the full solid angle in both UV-VIS-NIR and FTIR spectroscopic measurements^22^. For diffuse reflectivity, the polymer coating is placed on the reflection port of the integrating sphere in which all the backscattered light bounces off the integrating sphere surface several times until detected by the detector (Fig. 2a,b). For diffuse transmissivity, the polymer coating is placed on the front port of the integrating sphere, allowing all the light transmitted through the polymer coating to be received by the detector (Fig. 2c,d). It is noted that the average reflectivity in both the visible (λ, 0.4 μm to 0.8 μm) and NIR region (λ, 0.8 μm to 2.5 μm) gradually rises with increasing ϕ from 0 to 70%, while the diffuse transmissivity drops significantly with increasing ϕ in both regions. It is validated by the optical images of polymer coatings in Fig. 2e. As ϕ increases from 0 (left) to 70% (right), the surface changes opacity and becomes whiter. In the mid-IR region, ϕ dependence in both diffuse reflectivity and transmissivity is also observed between 2.5 μm and 6 μm, while it is not significant between 6 μm to 16 μm including the atmosphere’s transmission window. Using the measured diffuse reflectivity and transmissivity, the emissivity of polymer coatings with varying ϕ in solar region and mid-IR region is calculated and plotted in Fig. S2a, based on the sum of transmissivity, reflectivity and absorptivity being unity and assumption that emissivity is considered equal to absorptivity under Kirchhoff’s Law^23^. In Fig. S2b, the measured UV-VIS-NIR reflectivity of 70 vol% polymer coating with different thicknesses from 500 µm to 2500 µm are plotted. It can be seen that a thickness of 500 µm leads to a fairly high reflectivity while the reflectivity varies little when the thickness is higher than 750 µm. The high solar reflectivity (0.92) in the 70 vol% polymer coating is comparable to or exceeds many previous reported values^10,14,15,18^, which enables strong reflection of sunlight for possible radiative cooling applications and eliminates the need for using metallic reflectors reported in some previous designs^14,15^. We attribute the broadband high reflectivity to the broad distribution of the glass bubble diameter ranging from 1 µm to over 40 µm with the average of 8 µm. Based on our understanding supported by the Mie Scattering theory, we can expect that far smaller or larger glass bubbles won’t provide a high reflectivity in the solar wavelengths and that glass bubbles with a uniform size distribution may not provide a broadband reflectivity, which is essential for solar reflection and radiative cooling.

Apart from diffuse reflectivity, we also measured specular reflectivity for polymer coatings with varying ϕ and determine the refractive index values n(λ) and k(λ) using measured specular reflectivity and transmissivity values from the following expression^24^:

3 $${\rm{n}}({\rm{\lambda }})={\left[\frac{4r}{{(r-1)}^{2}}-{k}^{2}\right]}^{0.5}-\frac{r+1}{r-1}$$

4 $${\rm{k}}({\rm{\lambda }})=\frac{\alpha \lambda }{4\pi }$$

where α is the absorption coefficient and defined as ${\rm{\alpha }}=\frac{1}{d}\,\mathrm{ln}\left(\frac{1}{t}\right)$. Equations (3) and (4) clearly indicate that specular reflectivity (r) and diffuse transmissivity (t) are required as input parameters, but the values of reflectivity (R) and transmissivity (T) directly obtained from the spectrometer need to be corrected before incorporating them to calculate the optical parameters because some losses at the air-sample interfaces generally come into picture^24,25^. Using corrections stated elsewhere, r and t are related to R and T using Eqs. (5) and (6) below^25^:

5 $${\rm{r}}=\frac{2R}{1+{t}^{2}+\sqrt{{(1+{t}^{2})}^{2}-4{t}^{2}R(2-R)}}$$

6 $${\rm{t}}=\frac{2T}{{(1-r)}^{2}+\sqrt{{(1-r)}^{4}-4{T}^{2}{r}^{2}}}$$

**Figure 3:**
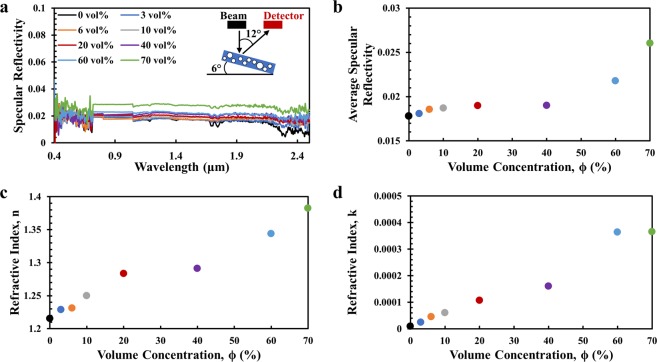
Refractive index values of polymer coatings with varying ϕ. (**a**) Specular reflectivity of polymer coatings with varying ϕ from 0 to 70% in the solar region and (**b**) the corresponding average specular reflectivity value from 0.4 μm to 0.8 μm. The inset in A shows that specular reflectivity is measured with an incident angle at 6° and detector angle at 12°; The average value of (**c**) real part, n(λ) and (**d**) imaginary part, k (λ) of refractive index from 0.4 μm to 0.8 μm for polymer coatings with varying ϕ from 0 to 70%. n(λ) and k(λ) are converted from measured reflectivity and transmissivity values from 0.4 μm to 2.5 μm for polymer coatings with varying ϕ from 0 to 70%.

The values of r and t are obtained by solving the equations through an iterative process until the value of r and t can repeat itself. Figure 3a shows the specular reflectivity as a function of wavelength from 0.4 μm to 0.8 μm for polymer coatings with varying ϕ. For specular reflectivity measurements, the incident beam angle and detector angle are set to 6° and 12°, respectively, as the inset in Fig. 3a shows. It is observed that specular reflectivity increases gradually with increasing ϕ, which agrees with what we found in diffuse reflectivity measurements but with smaller increments. We observe some noise in the measured values of specular reflectivity in the visible region due to the inherent uncertainty of the spectrometer. Thus, the average specular reflectivity between 0.4 μm to 0.8 μm as a function of volume concentration ϕ is plotted in Fig. 3b and it increases from 0.018 to 0.026 for increasing ϕ from 0 to 70%. The values of refractive index n(λ) and k(λ) as a function of wavelength has been determined using Eqs. (3) to (6). The calculated n and k values are functions of wavelength possibly because the refractive index of two components, PDMS and SiO_2_, are both functions of wavelength. Similar to both the specular reflectivity and the diffuse reflectivity, n (λ) and k(λ) both increase with increasing ϕ. Average values of n(λ) and k(λ) from 0.4 μm to 0.8 μm for varying ϕ are plotted in Fig. 3c,d. The average n and k values increase from 1.21 to 1.38 and from 9.576 × 10^−6^ to 3.655 × 10^−4^ respectively, with increasing ϕ from 0 to 70%. The calculated refractive index values indicate how light propagates through the polymer coatings with varying ϕ. With a larger refractive index value for larger ϕ, the light travels slower, which correspondingly causes more changes in the direction of light propagation within polymer coatings. This leads to increased diffuse reflectivity or specular reflectivity and decreased diffuse transmissivity for polymer coatings with increasing ϕ as we report. We use the computation based on rigorous coupled-wave analysis (RCWA)^26–28^ to verify the ϕ dependence on optical properties of polymer coatings and the computation results agree well with the measured optical properties of the polymer coatings (detailed in Section S3 and Figs. S3 to S4 in the Supplementary Information).

### Section 3. Thermal analysis and outdoor temperature measurement

**Figure 4:**
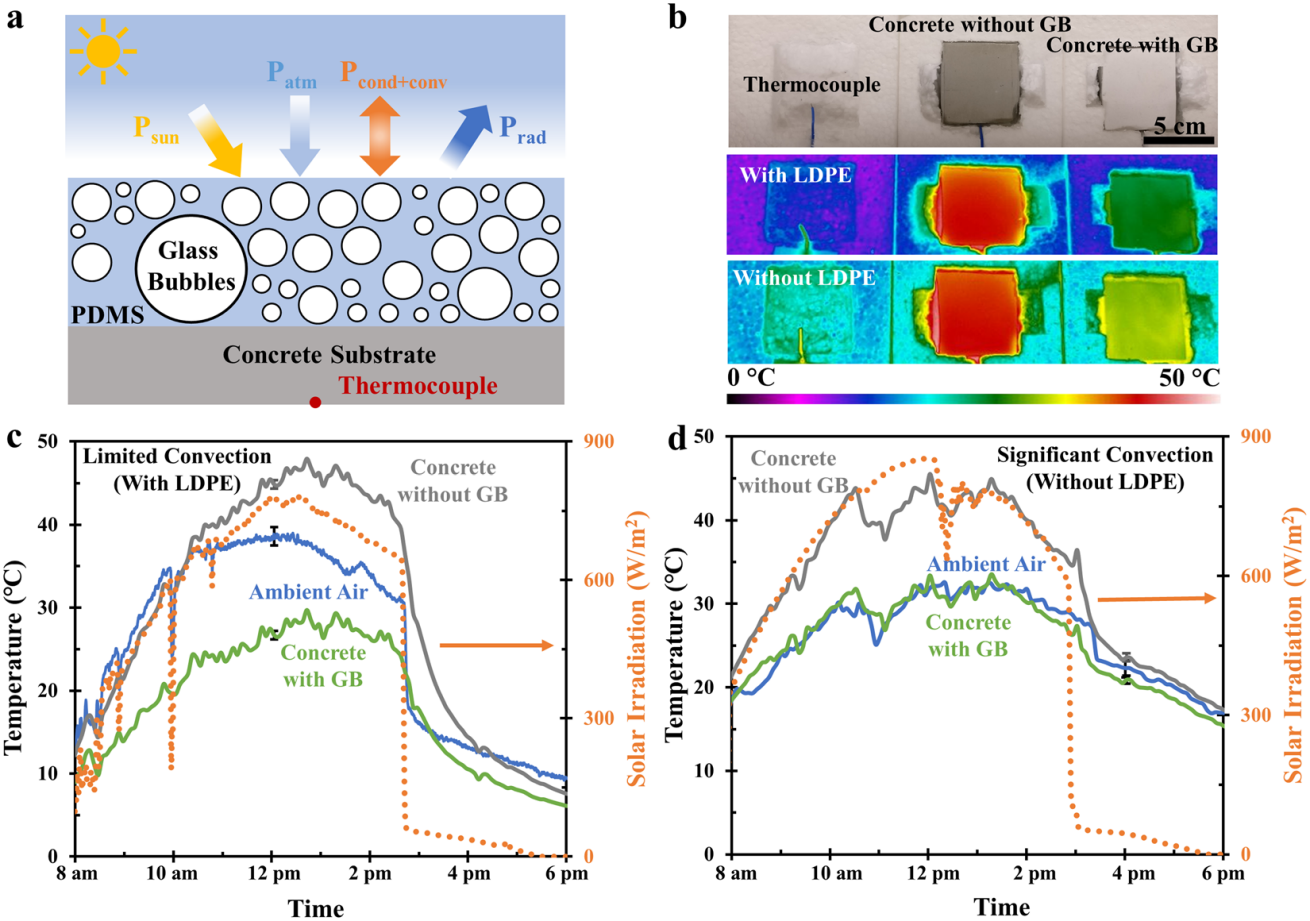
Thermal performance of 70 vol% polymer coating. (**a**) The schematic of the thermal analysis of a polymer coating in the ambient environment. The thermocouple in the schematic shows the location of thermocouples measuring sample temperatures during the measurements; (**b**) The optical image of the rooftop temperature measurement apparatus with or without a 25 μm-thick layer of low-density-polyethylene (LDPE) film as the wind shield. In the optical image, the left is the thermocouple used for measuring ambient air temperature. The middle is the concrete block of 2 inch × 2 inch × 0.5 inch with a same-area 2 mm-thick PDMS on top (denoted as ‘concrete without glass bubble (GB)’) and the right is the concrete block with a same-area 2 mm-thick 70 vol% polymer coating on top (denoted as ‘concrete with GB’); Rooftop temperature measurement data from 8 am to 6 pm for ambient air (blue), concrete without GB (grey) and concrete block with GB (green) (c) with the LDPE film covered on the top as the wind-shield and (d) without the LDPE film during the measurement. The temperature data in c and d was measured on Feb. 19^th^, 2020 and Mar. 3^rd^, 2020, respectively.

The combination of high solar reflectivity and mid-IR emissivity we have obtained from the spectroscopy is promising for radiative cooling applications. The measured values of 70 vol% polymer coating are used in the temperature predictions based on thermal analysis in the ambient environment (detailed in Fig. 4a and **Section S4** in the Supplementary Information). The prediction in Fig. S5a shows that the concrete temperature with 70 vol% polymer coating is 5.3 °C lower than the ambient air at noon due to the unique combination of high solar reflectivity and mid-IR emissivity, assuming a steady ambient environment, a representation daily weather data in the summer^29^, a constant conductive and convective heat transfer coefficient h=10 W/m^2^K and a peak solar irradiance^30^ of 875 W/m^2^ at 12 pm (shown in Fig. S5a). It is noticed that the bare concrete block without 70 vol% polymer coating is predicted to be 35 °C higher than the ambient air at noon. In terms of the relation between temperature and heat flux (Fig. S5b), the prediction shows that the maximum temperature drop can reach 5.3 °C, while the cooling power at the peak solar irradiance is 78.2 W/m^2^, which is around half of the theoretical maximum cooling power (147.9 W/m^2^) as the prediction indicates. This remarkable cooling performance is comparable to many previously reported works whose average daytime cooling power varies from 40 W/m^2^ to 96 W/m^2^ ^10,14,15,18,31^, or from 42 W/m^2^ to 109 W/m^2^ when we evaluate these radiative cooling materials under same cooling power prediction conditions as our polymer coating.

We conduct the temperature measurements to demonstrate the surface cooling capability of the 70 vol% polymer coating, as shown in Fig. 4b. A 2 inch × 2 inch × 0.5 inch concrete block with a same-area 2-mm thick PDMS on top (denoted as ‘concrete without GB’ in Fig. 4b, ‘GB’ means glass bubbles) and another same-size concrete block with a 2 mm-thick 70 vol% polymer coating on top surface (denoted as ‘concrete with GB’ in Fig. 4b) are used for a comparative study. The samples are placed inside the insulation Styrofoam with a low thermal conductivity of 0.063 W/(m·K)^32^ to minimize conductive heat loss. The temperature data in Fig. 4c,d is obtained using the IR camera with an uncertainty of ±0.5 °C and thermocouples with an uncertainty of ±1.1 °C. The solar irradiation corresponding to the right y-axis is measured using a pyranometer for reference. With a 2 mm-thick layer of 70 vol% polymer coating covered on top surface, the concrete block exhibits a sub-ambient cooling of 9.6 °C at peak solar irradiance of 780 W/m^2^ when a 25 µm-thick low-density-polyethylene (LDPE) film lies above the box as an IR-transparent wind shield (Fig. 4c). The temperature reduction is also apparent in the IR image at 1 pm, in which the concrete with 70 vol% polymer coating in the right cools around 20 °C than concrete block with PDMS in the middle. We’ve confirmed that the temperatures of the bare concrete sample and the PDMS coated concrete sample with no glass bubbles are nearly identical (See Fig. S6 in the Supplementary Information). We also conduct the outdoor temperature measurement without the LDPE film to simulate the realistic exposed surfaces of buildings (Fig. 4d). Compared with concrete with PDMS, the concrete block with the 70 vol% polymer coating shows a comparable temperature as ambient air. This phenomenon is also apparent from the IR image at 12 pm. The abrupt drops of measured solar irradiance at 10 am and 12 pm is possibly due to the clouds in the sky, while the drop at approximately 3 pm is due to the shadows casted on the pyranometer. The mismatch between temperature prediction in Fig. S5a and temperature measurements in Fig. 4c,d is due to that the convective and conductive heat transfer coefficient h is always changing during the whole measurement slot and it deviates from the constant value we use in the temperature prediction. Considering concrete is a widely used building material, the outdoor temperature measurements both with and without wind-shielding LDPE film exemplify the surface radiative cooling capability of the 70 vol% polymer coating, thus enabling substantial energy savings and large-scale realistic application in surface radiative thermal management for buildings, spacecraft or wearables.

### Section 4. Building energy consumption analysis

**Figure 5:**
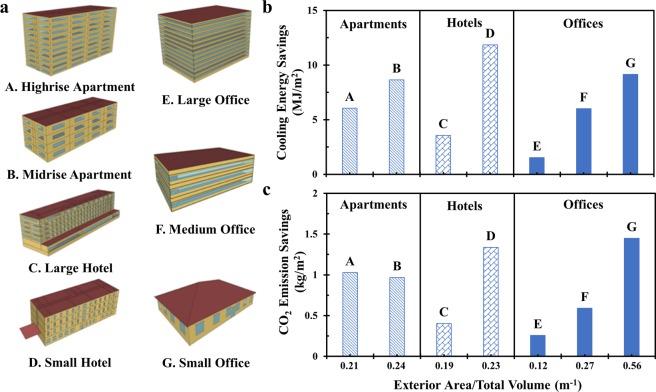
Annual cooling energy savings and CO_2_ emission savings by modifying the reference building types with 70 vol% polymer coating. Here, Department of Energy (DOE) developed reference building models and ASHRAE Standard 90.1 prototype building models (New Construction after 2004) including highrise apartment (A), midrise apartment (B), large hotel (C), small hotel (D), large office (E), medium office (F) and small office (G) located in Los Angeles are used for the energy consumption analysis. The building structures are modified by adding a 2 mm-thick layer of 70 vol% polymer coating for roofing and siding on the exterior surfaces. (**a**) 3D models of the building models from highrise apartment (A) to small office (G); (**b**) Annual cooling energy savings per area for different building types, showing that building types with larger ratio of exterior area over total volume saves more cooling energy per area per year; (**c**) Annual CO_2_ emission savings per area associated with electricity consumption reduction for different building types.

The surface cooling capability of the 70 vol% polymer coating when applied onto the surfaces of generally used building materials motivates us to seek out the potential energy savings of using such polymer coatings on the exterior surfaces of commercial and residential buildings. Among different building types and representative cities in various climate zones provided by the US Department of Energy (DOE) commercial reference building database^33^ and The American Society of Heating, Refrigerating and Air-Conditioning Engineers (ASHRAE) Standard 90.1 prototype building database^34^, seven different building models (New Construction after 2004) located in Los Angeles are selected, representing common commercial and residential buildings and providing comparison within the same category. Los Angeles is selected because it is a representative city with hot climate and large population. We use A, B, C, D, E, F and G to denote highrise apartment, midrise apartment, large hotel, small hotel, large office, medium office and small office, respectively. Detailed dimensional information of these building models is listed in Table S2 and three-dimensional models are provided in Fig. 5a. The total exterior surface area for seven building models varies much. The largest surface area of 15747 m^2^ is provided by large office model, while small office model only has a surface area of 880 m^2^, which is smallest among seven different models. We use the building energy simulation software EnergyPlus developed by DOE to solve the governing heat balance equations with an hourly time step manner over a whole year. The simulation assumes that the internal air temperature is set to 24 °C and the external air temperature are determined by hourly Typical Meteorological Year (TMY3) weather data^35^, which are used as the input boundary conditions for the governing equations. The building models are directly used to establish the reference energy consumption patterns and then modified by adding a 2 mm-thick layer of 70 vol% polymer coating onto the exterior surfaces of roofs and walls to generate the modified energy consumption patterns. Detailed material properties of 70 vol% polymer coating are specified in Table S3.

We calculated the annual cooling energy savings for seven selected building types from A to G located in Los Angeles via comparing annual cooling energy generated by reference building types and modified building types (Fig. S7a). To eliminate the influence of different building area and surface area for different building models, the annual cooling energy savings per area are shown and compared in Fig. 5b. Seven building types are classified into three categories: apartments, hotels and offices. We use the ratio of exterior surface area (including roofing area and siding area) over total volume of the building models as the x-axis in Fig. 5. We find that annual cooling energy savings of the modified building models ranges from 1.52 MJ/m^2^ to 11.85 MJ/m^2^ (0.42 kWh/m^2^ to 3.29 kWh/m^2^). It is also noticed that a lower-rise apartment, hotel or office has a larger ratio of exterior area over total volume and saves more annual cooling energy per area or has a larger reduction in annual cooling energy when 70 vol% polymer coating is covered on the exterior surface. For instance, compared with highrise apartment with 9 floors and a ratio of 0.21 m^−1^, the midrise apartment with 4 floors has a higher ratio (0.24 m^−1^) of exterior area over total volume and saves 8.6 MJ/m^2^ cooling energy per year, which is 2.5 MJ/m^2^ higher than annual cooling energy savings of highrise apartment. The polymer-coating-modified midrise apartment saves 27.5% of annual cooling energy compared to reference midrise apartment, while polymer-coating-modified highrise apartment saves 19.7% annual cooling energy compared to reference one. The same relationship is found between large hotel and small hotel, as well as large, medium and small offices. Besides annual cooling energy savings, we also carry out the analysis in terms of annual heating energy, electricity consumption and total energy. Total energy includes heating energy and electricity consumption, while electricity consumption consists of cooling energy, lightning, equipment electricity consumption, fan energy and refrigeration, etc. Figs. S7b to S7d summarize the annual heating energy, annual electricity consumption and annual total energy for both reference and modified building models. It is observed that annual heating energy is increased for all building types possibly because the polymer coating on the exterior surface lower the indoor air temperature during the cold days^31^. As for the annual electricity consumption and annual total energy, all seven building types exhibit a slight reduction despite of gained annual heating energy. Annual CO_2_ emission savings associated with annual electricity consumption reduction for seven 70 vol%-polymer-coating-modified building models are also estimated and presented in Fig. 5c. We find that the annual CO_2_ emission savings per area ranges from 0.26–1.45 kg/m^2^, showing the excellent promise in reducing the greenhouse gas emission. Energy analysis in other locations such as Phoenix, Seattle or Chicago are also conducted and the annual cooling energy savings increase with a hotter and dryer climate, with the same input material properties of 70 vol% polymer coating specified in Table S3 and local TMY3 weather conditions. The building energy savings analysis exemplifies the significant cooling energy savings for common commercial and residential buildings, especially for low-rise buildings with a larger ratio of exterior area over total volume.

Our cool white polymer coating exhibits great advantages over existing benchmarked approaches such as commercial white paints regarding building cost savings, greenhouse gas emission savings and material costs. Our analysis of representative buildings in Los Angeles shows that our cool white polymer coating will lead to annual cost savings of $0.05 – $0.58/m^2^ (Fig. S8a), while commercial white paints provide savings of $0.03 – $0.31/m^2^, with detailed input material properties of commercial white paints^11,18^ specified in Table S3. For the annual CO_2_ emission savings, our analysis predicts our cool white polymer coating to save 0.26–1.45 kg/m^2^ (Fig. 5c), while commercial white paints are expected to save 0.14–0.76 kg/m^2^ (Fig. S8b**)**. The large NIR reflectivity of 0.82 and UV-resistant property (Detailed in **Section S6** and Fig. S9 in the Supplementary Information) make it more attractive for building applications compared to commercial white paints. The advantages of the polymer coating over white paints pave the way for potential applications in surface radiative cooling for buildings and spacecraft since they both suffer from heavy UV irradiation and UV rays damage the modern buildings gradually as reported before^36^. Additionally, it is noted that PDMS is not the only choice for the matrix material. Our polymer coating system is compatible with a wide variety of polymers that have similar optical properties such as poly(methyl methacrylate)^37^ and poly(vinylidene fluoride-co-hexafluoropropene)^12^ (Fig. S10). In terms of techno-economic evaluation, cost per square meter or cost per cooling power is an important factor to determine whether the solution is cost-effective for realistic applications. Based on the representative bulking price on market and a thickness of 500 µm (detailed in Section S6 in the Supplementary Information), our techno-economic analysis presents our cool white polymer coating to be very attractive in terms of $0.39/m^2^ and $0.005/W compared to commercial white paints^11^, which have $0.48/m^2^ and $0.012/W, or even more superior compared to the state-of-the-art radiative cooling material^14^ with $2.49/m^2^ and $0.027/W in cost.

To sum up, the low-cost and scalable cool white polymer coating with a controlled concentration of glass bubbles can have the solar reflectivity of 0.92 and the mid-IR emissivity of 0.85, which leads to significant radiative cooling. The outdoor temperature measurement shows that 70 vol% polymer coating on a concrete surface helps to achieve a maximum temperature drop of 25 °C compared to the bare concrete block and exhibits a maximum sub-ambient cooling of 9 °C during the daytime with limited convective and conductive heat transfer. The building energy consumption analysis based on seven common building models in Los Angeles indicates that the annual cooling energy savings could be achieved from 2 MJ/m^2^ to 12 MJ/m^2^ by using the 70 vol% polymer coating on the exterior surface and the savings are more significant for higher surface area-to-volume buildings. The associated annual cost savings and annual CO_2_ emission savings for representative buildings in Los Angeles are predicted to range from $0.05/m^2^ to $0.58/m^2^ and 0.26 kg/m^2^ to 1.45 kg/m^2^, respectively. The techno-economic analysis shows that the material costs for our cool white polymer coatings is estimated to be $0.39/m^2^ and $0.005/W. This work opens the possibility of using such polymer-based coatings in large-scale surface cooling for modern buildings and pave the way towards energy-efficient buildings to reduce energy consumption. Furthermore, this work provides a promising solution to resolve cooling issues for buildings with insufficient air conditioning systems, and address the global concern of the record-breaking heat waves occurred in recent years.

## Methods

### Fabrication of polymer coatings

The silicone elastomer base and curing agent (Sylgard 184, from Dow Corning) is mixed with a 10:1 weight ratio in a vial to make polydimethylsiloxane (PDMS). After thoroughly stirring, it is added into a pre-weighted amount of glass bubbles (iM16K, from 3 M) for preparation of polymer coatings with varying volume concentrations (ϕ) of glass bubbles. After being completely blended and degassed, the mixture is cast onto a substrate and dried under ambient conditions for 24 hours. It is then transferred into an oven for 2-hours 60 °C heating under vacuum conditions.

### Mass density measurements

The theoretical mass density of the polymer coating is calculated by the known density of the PDMS and glass bubbles. The measured mass density is equal to the measured weight of the polymer coating divided by the measured volume which is length times width times thickness. The areal density equals to the measured mass density times the measured thickness of the polymer coating.

### Optical spectroscopy

The optical properties of polymer coatings in the visible and NIR region are characterized by Ultraviolet-Visible-NIR (UV-VIS-NIR) spectrometer (Cary 7000, Agilent and Jasco V670 coupled with a 60 mm integrating sphere, Jasco Technology) in the wavelength range from 0.4 μm to 2.5 μm. The diffuse reflectivity and transmissivity measurements are calibrated with a standard white body. The optical properties of polymer coatings in mid-IR region are characterized by Fourier-transform infrared (FTIR) spectrometer (Nicolet 6700, Thermo Scientific) with an integrating sphere (Mid-IR IntegratIR^TM^, Pike Technologies). The emissivity is calculated based on the sum of the transmissivity, reflectivity and absorptivity being unity and Kirchhoff’s Law^23^ assuming that emissivity is considered equal to absorptivity. The optical measurements have an inherent uncertainty of ±0.003 for the properties ranging from 0 to 1 and the wavelength uncertainty of the spectrometers is ±0.3 nm at a standard room temperature of 25 °C.

### Scanning electron microscopy

Scanning electron microscopy images of the polymer coatings and glass bubbles are taken using a Philips XL-30 FEG scanning electron microscope (SEM).

### Outdoor temperature measurements

The temperature measurement is conducted on the rooftop of the Engineering Tower at University of California, Irvine (UCI). The concrete block with a dimension of 2 inch × 2 inch × 0.5 inch and another same-size concrete block with a 2 mm-thick layer of 70 vol% polymer coating on top surface are used for a comparative study. The concrete blocks with and without 70 vol% polymer coating are placed inside the insulation Styrofoam with a low thermal conductivity of 0.063 W/(m·K)^32^, which is stabilized in a box with aluminum foil coated on the outside wall and white paper coated on the inside wall. The box with and without the wind-shield low-density-polyethylene (LDPE) film are used to simulate different surface conditions where convective heat transfer is minimized or presented. The infrared (IR) camera (FLIR, A655sc) and thermocouples (K-type, Omega) attached to a thermometer (RDXL12SD, Omega) are both used to measure the temperatures and calibrate each other. The resolution of the K-type thermocouple is 0.1 °C with an inherent measurement uncertainty of ±1.1 °C at a temperature region from 0 °C to 55 °C, while the resolution of the FLIR IR camera is 0.03 °C with an inherent uncertainty of ±0.5 °C for a measurement range of 0 °C to 70 °C. The temperature of ambient air is also measured using K-type thermocouples for comparison with predicted ambient air temperature and demonstration of temperature influence of different samples.

### Synchrotron characterization

The tomography measurement (Nano CT) is done at Irvine Materials Research Center (IMRI). The effective pixel size is 1.02 μm × 1.02 μm. Tomography data reconstruction is done with Simpleware ScanIP, a three-dimensional (3D) segmentation and processing software developed by Synopsys Inc. The structures are thresholded and segmented from the tomographic reconstructed data and the glass bubbles’ size distribution analysis is done with ImageJ, ‘3D Objects Counter’ plugin.

## Supplementary information

Supplementary Information.
