# Supplementary material for: Cool White Polymer Coatings based on Glass Bubbles for Buildings
Source: Sci Rep. 2020 Apr 20;10:6661. doi: 10.1038/s41598-020-63027-2 (PMC7170890; doi:10.1038/s41598-020-63027-2)
Supplement: Supplementary file 1 — Supplementary Information. [file 41598_2020_63027_MOESM1_ESM.docx]

**Supplementary Information**

Cool White Polymer Coatings based on Glass Bubbles for Buildings

Xiao Nie^1,†^, Youngjae Yoo^1,2,†,*^, Hasitha Hewakuruppu^1^, Jonathan Sullivan^1^, Anirudh Krishna^1^, Jaeho Lee^1,*^

^1^Department of Mechanical and Aerospace Engineering, University of California, Irvine, CA 92617, USA

^2^Advanced Materials Division, Korea Research Institute of Chemical Technology, Daejeon 34114, Korea

^†^X.N. and Y.Y. contribute equally to this work.

^*^Corresponding author: jaeholee@uci.edu, yjyoo@krict.re.kr

**Section S1. Preparation, characterization and density analysis**

We prepared the polymer coatings by integrating glass bubbles inside the PDMS matrix and controlling the volume concentrations (ϕ) from 0 to 70%. We conducted the size analysis of the as-purchased glass bubbles using SEM images in multiple observation locations. The results are shown in **Fig. S1a** to **S1d**, and the corresponding SEM images are presented in the inset. From the results we notice that the diameters of glass bubbles are near-normally distributed, with a broad distribution range from 1 μm to roughly 40 μm and center at around 8 μm. The mean diameters of glass bubbles in these four different SEM images are 10.9 μm, 9.7 μm, 10.4 μm and 9.9 μm, respectively. After blended with PDMS, we conducted the nano Computed Tomography (CT) analysis and the result is shown in **Fig. S1e**. Nano CT uses X-rays to detect the cross-sections of the three-dimensional polymer coatings. The reconstructed virtual model using cross-sections images with the help of a three-dimensional segmentation and processing software is shown in the **inset** of Fig. S1e. It is noticed that for over 95% glass bubbles the diameter ranges from 1 μm to 50 μm and the mean value is 12 μm. After mixing glass bubbles with PDMS, the lightweight polymer coatings with varying ϕ from 0 to 70% are prepared and the density were measured and compared with theoretical values. Taking PDMS density (0.965 g/cm^3^ or 965 kg/m^3^) and glass bubble density (0.460 g/cm^3^ or 460 kg/m^3^) into consideration, we obtain the theoretical mass density (ρ) of the polymer coating as a function of ϕ of glass bubbles as follows:

$$\begin{aligned} \rho\left( \frac{kg}{m^{3}} \right)=\frac{\left( 100-\phi\right)}{100}*965+\frac{\phi}{100}*460\#\left( S1 \right) \end{aligned}$$

As shown in **Table S1**, the measured mass density of the polymer coating drops from 905 kg/m^3^ to 681 kg/m^3^. Regarding the areal density, 3 vol% polymer coating with a thickness of 330 μm results in 0.306 kg/m^2^, while 70vol% polymer coating with a thickness of 1300 μm leads to 0.885 kg/m^2^.

**Section S2. Optical properties of polymer coatings**

We used ultraviolet-visible-near infrared (UV-VIS-NIR) spectrometer and Fourier-transform infrared (FTIR) spectrometer to obtain the optical properties of polymer coatings with varying ϕ from 0 to 70% in the wavelength range from 0.4 μm to 16 μm. The reflectivity and transmissivity are measured directly, while emissivity is calculated based on the sum of the transmissivity, reflectivity and absorptivity being unity and Kirchhoff’s Law^1^ assuming that emissivity is considered equal to absorptivity. Reflectivity and transmissivity data for varying ϕ from 0 to 70% is presented in **Fig. 2**, while emissivity is plotted in **Fig. S2a**. In **Fig. S2b**, the measured UV-VIS-NIR reflectivity of 70vol% polymer coating with different thicknesses from around 500 µm to 2500 µm are plotted. It can be seen that a thickness of 500 µm leads to a fairly high reflectivity while the reflectivity varies little when the thickness is higher than 750 µm.

**Section S3. Rigorous coupled-wave analysis (RCWA)**

Rigorous coupled-wave analysis (RCWA) solves the Maxwell’s equations analytically and presents the results as an infinite series summation.

The refractive index values for varying ϕ from 0 to 70% were calculated using measured specular reflectivity values and diffuse transmissivity values between 0.4 μm to 2.5 μm. The equations (3) to (6) in **Section 2** were utilized and the results are presented in **Fig. 3**. To verify the calculated refractive index values, we performed a slab assumption using RCWA. The slab is assumed 2 × 2 × 600 μm^3^, with periodic boundary conditions in x and y directions. With the calculated refractive index values for varying ϕ as the input parameters, we observe an increase in computed average reflectivity and a decrease in computed average transmissivity from 0.4 μm to 2.5 μm with increasing ϕ from 0 to 70% (**Fig. S3a** and **S3b**), which is in agreement with the measured specular reflectivity values and diffuse transmissivity values we obtained from UV-VIS-NIR spectrometer.

We use the rigorous coupled wave analysis (RCWA) method to evaluate volume concentration dependence on optical properties of polymer coatings. RCWA^2^ handles solutions to Maxwell’s equations and topological variations. Direct results from RCWA yield scattering matrices in the forward and reverse directions, from which transmissivity and reflectivity are computed. We developed a custom RCWA code to model the polymer coating to a three-dimensional periodic unit cell. The optical properties of PDMS^3^ and SiO_2_^4^ reported previously are used for the simulation of polymer coatings. The normally distributed diameter of glass bubbles is used as the geometric parameter in our computation. The emissivity is assumed identical to the absorptivity according to Kirchhoff’s Law^5^, with the sum of absorptivity, reflectivity and transmissivity being unity. A unit cell with the dimension of 50^3^ μm^3^ or 75^3^ μm^3^ and a normal diameter distribution of glass bubbles (**Fig. S3c**) are used for polymer coatings with varying ϕ from 0 to 40%. Higher ϕ can be achieved if forced but it causes significant deviation from normal diameter distribution for glass bubbles, so we prefer computations with lower ϕ in this work. The 75^3^ μm^3^ unit cell is used to model 0 vol%, 3 vol% and 6 vol%, while the 50^3^ μm^3^ unit cell is used to present 10vol%, 20 vol% and 40 vol%. The glass bubbles inside the unit cell are layered into stacked layered structures (**Fig. S3d**) and the unit cell was stacked in the z direction for the desired thickness (**Fig. S3e**). For our RCWA computation, the thickness is set to 600 μm for all ϕ.

The schematics of unit cells with varying ϕ from 0 to 40% is shown in **Fig. S4a** and the cross-sectional view of one single glass bubble is presented in **Fig. S4b**. The average values of computed reflectivity (**Fig. S4c**) and transmissivity (**Fig. S4d**) show the volume concentration dependence in the solar region. When ϕ goes up from 0 to 40%, the average reflectivity between 0.4 μm and 2.5 μm increases from 0.05 to 0.39, while the average transmissivity from 0.4 μm to 2.5 μm decreases from 0.86 to 0.55. In Fig. S4c and S4d, both average computed reflectivity and transmissivity are compared with the average measured values. It is observed that the computation results agree well with the measured optical properties of the polymer coatings. The RCWA computation has also predicted the optical properties in the mid-IR range and shows that the presence of glass bubbles does not make significant impact on wavelengths between 8 µm and 13 μm (**Fig. S3f**), which agrees with IR thermography measurement results and is relevant to radiative thermal characteristics.

**Section S4. Thermal Analysis and outdoor temperature measurement**

The polymer coating is considered at an area A with some certain temperature T, whose spectral and angular emissivity is ε (λ, θ). When it is exposed to steady daytime ambient environment, it is subject to both solar irradiation and atmospheric thermal radiation. Assuming the ambient temperature is T_amb_, the net cooling power of the polymer coating at temperature T is expressed as^6–8^:

$$\begin{aligned} P_{cooling}\left( T \right)=P_{rad}\left( T \right)-P_{sun}-P_{atm}\left( T_{atm} \right)-P_{con} \#\left( S2 \right) \end{aligned}$$

In the right-hand side of equation (S2), the first term $P_{rad}(T)$ is the power radiated by the polymer coating:

$$\begin{aligned} P_{rad}\left( T \right)=A\int d\Omega cos\theta\int_{0}^{\infty} d\lambda I_{BB}\left( T,\lambda\right)\varepsilon\left( \lambda,\theta\right)\#\left( S3 \right) \end{aligned}$$

In the equation, $\int d\Omega=2\pi\int_{0}^{\pi/2} d\theta sin\theta$ is the angular integral over a hemisphere and $I_{BB}\left( T,\lambda\right)=\frac{2hc^{2}}{\lambda^{5}}\cdot\frac{1}{e^{hc/(\lambda k_{B}T)}-1}$ is the spectral radiance of a perfect blackbody at the temperature T. Here, h is the Planck’s constant, c is the speed of light and k_B_ is the Boltzmann constant.

The second term $P_{atm}(T_{atm})$ presents the incident solar power absorbed by the polymer coating and the solar illumination is given by $I_{AM1.5}(\lambda)$, the AM 1.5 spectrum.

$$\begin{aligned} P_{sun}=A\int_{0}^{\infty} d\lambda\varepsilon\left( \lambda,\theta\right)I_{AM1.5}\left( \lambda\right)\#\left( S4 \right) \end{aligned}$$

The third term $P_{atm}(T_{atm})$ and the fourth term $P_{con}$ in equation (S2) is the incident atmospheric thermal radiation power and the power loss due to the conduction and convection, respectively:

$$\begin{aligned} P_{atm}=A\int d\Omega cos\theta\int_{0}^{\infty} d\lambda I_{BB}\left( T,\lambda\right)\varepsilon\left( \lambda,\theta\right)\varepsilon_{atm}\left( \lambda,\theta\right)\#\left( S5 \right) \end{aligned}$$

$$\begin{aligned} P_{con}=Ah_{c}\left( T_{atm}-T \right)\#\left( S6 \right) \end{aligned}$$

Here $h_{c}=h_{cond}+h_{conv}$ represents the sum of the non-radiative heat transfer coefficient capturing conductive and convective heating because of the contact between the polymer coating and external surfaces and ambient air adjacent to the film. **Fig. S5a** represents the temperature prediction of the concrete block with and without the 70vol% polymer coating applied onto the surface during the daytime, assuming a steady ambient environment, a representative daily weather data in summer^9^, a constant conductive and convective heat transfer coefficient h=10 W/m^2^K and a peak solar irradiance^10^ of 875 W/m^2^ at 12 pm. The prediction shows that the concrete temperature with 70 vol% polymer coating is 5.3°C lower than the ambient air at noon due to the unique combination of high solar reflectivity and infrared emissivity. It is noticed that the bare concrete block without 70vol% polymer coating is predicted to be 35 °C higher than the ambient air at noon. **Fig. S5b** shows a relationship between sub-ambient cooling temperature of 70 vol% polymer coating and cooling power it offers, assuming a constant heat transfer coefficient h= 10 W/(m^2^K) and an ambient air temperature of 25 °C. The prediction shows that the maximum temperature drop can reach to 5.3 °C for 70 vol% polymer coating, while the cooling power at the peak solar irradiance of 875 W/m^2^ is 78.2 W/m^2^. **Fig. S5c** and **S5d** presents two temperature measurement data obtained on July 11^th^, 2019 and June 28^th^, 2019 respectively, showing temperatures of bare concrete, concrete with 70 vol% polymer coating and ambient air with (Fig. S5c) and without (Fig. S5d) a 25-µm-thick low-density-polyethylene (LDPE) as the wind shield during the measurement. The radiative cooling effect is significant because the concrete with 70 vol% polymer coating cools around 20 °C compared to bare concrete. **Fig. S6** demonstrate that temperatures of bare concrete and concrete with PDMS are almost the same during the measurement period from 12:30 pm to 2:30 pm on Mar. 8^th^, 2020.

**Section S5. Building energy consumption analysis**

We carried out the annual energy consumption analysis based on seven different building models in Los Angeles provided by Department of Energy commercial reference building database^11^ and The American Society of Heating, Refrigerating and Air-Conditioning Engineers (ASHRAE) Standard 90.1 prototype building database^12^ (New Construction after 2004). We used A, B, C, D, E, F and G to represent highrise apartment, midrise apartment, large hotel, small hotel, large office, medium office and small office, respectively. The dimensional information of seven building models are listed in **Table S2**. We observe that a low-rise apartment, hotel or office has a larger ratio of exterior area over total volume. The reference building models from the database are directly utilized to generate reference energy consumption patterns and they are modified by adding a 2mm-thick layer of 70vol% polymer coating onto the exterior surfaces of roofs and walls to generate the modified energy consumption patterns. The detailed input material properties including thermal and optical properties of the 70vol% polymer coating are listed in **Table S3**. We also conducted the analysis in terms of annual cooling energy, heating energy, electricity consumption and total energy, which are presented in detail in **Fig. S7**. Total energy includes heating energy and electricity consumption, while electricity consumption consists of cooling energy, lightning, equipment electricity consumption, fan energy and refrigeration, etc.  Our analysis of representative buildings in Los Angeles shows that our cool white polymer coating will lead to annual cost savings of $0.05 – $0.58/m^2^ (**Fig. S8a**), while commercial white paints provide savings of $0.03 – $0.31/m^2^. For the annual CO2 emission savings, our analysis predicts our cool white polymer coating to save 0.26 – 1.45 kg/m^2^ (**Fig. S8b**), while commercial white paints are expected to save 0.14 – 0.76 kg/m^2^.The detailed input material properties of the white paint^13,14^ are listed in **Table S3** as well.

**Section S6. High NIR reflectivity, UV-damage-free property and techno-economic analysis**

When compared with commercially available pigment-embedded (usually TiO_2_) white paint^13^, the 70 vol% polymer coating presents a comparable reflectivity in visible region and a much higher reflectivity in the NIR region. As **Fig. S9a** shows, the reflectivity of the TiO2-based white paint drops quickly beyond visible region, both in wavelength ranges smaller than 0.45 μm and larger than 0.8 μm. Meanwhile, the 70 vol% polymer coating has little absorption in the ultraviolet to blue wavelengths and the reflectivity maintains above 0.8 up to 2.3 μm. In addition, we conducted UV damage tests in which the beam of UV light at 300 nm with the power of 11.9 mW was irradiated onto the 70 vol% polymer coating for 120 hours and the solar reflectivity shows no change and still maintains high (**Fig. S9b**).

We conducted the techno-economic analysis based on the average bulking prices on market. By using a 70% volume concentration of glass bubbles and a thickness of 500 µm, we show that the cost of 70 vol% polymer coating is only $0.39/m^2^ and $0.005/W. A cooling power of 78.2 W/m^2^ is used in the calculation here. In Fig. S2b we show that 500 µm already leads to a fairly high solar reflectivity. If a thickness of 750 µm is used, the cost of 70 vol% polymer coating is $0.58/m^2^ and $0.007/W. Using the price information from the same market source, we found that commercial white paint costs $0.48/m^2^ and $0.012/W and the state-of-the-art radiative cooling material^15^ leads to a cost of $2.49/m^2^ and $0.027/W. For this calculation we take a cooling power of 40 W/m^2^ for white paint^16^ and 96 W/m^2^ for the state-of-the-art radiative cooling materials as reported previously. The techno-economic analysis presents our polymer coating very attractive in terms of cost per area or cost per cooling power it provides.


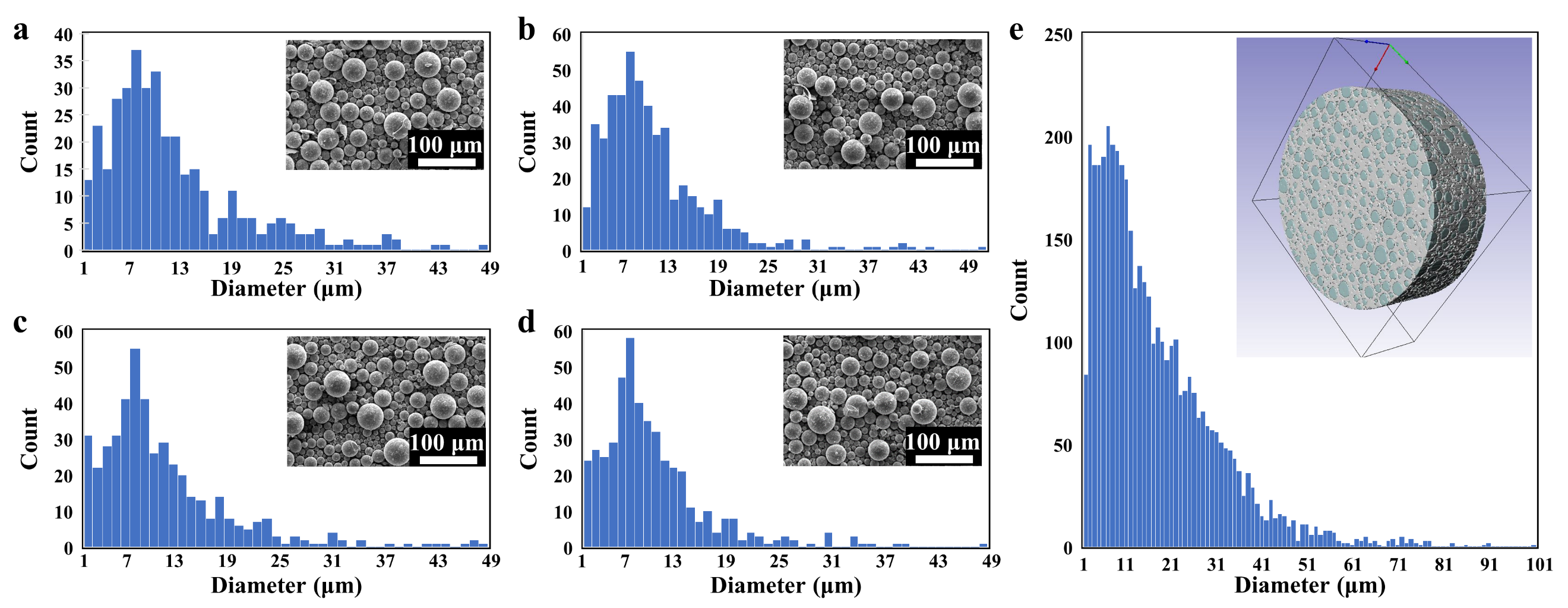


**Fig. S1. Diameter distribution analysis of glass bubbles before mixed with PDMS from SEM images and after mixed with PDMS from nano CT analysis.** (**a**-**d**) The corresponding diameter distribution of glass bubbles from top-down view SEM image in the inset. All the diameter distributions agree well, with similar diameter ranges and mean values. For all SEM images, the mean values of diameters are 10.9 μm, 9.7 μm, 10.4 μm and 9.9 μm, respectively; (**e**) The diameter distribution of the glass bubbles after blended with PDMS using nano CT analysis. The inset is the three-dimensional model of the 70 vol% polymer coating rebuilt from cross-sectional images obtained from nano CT analysis.


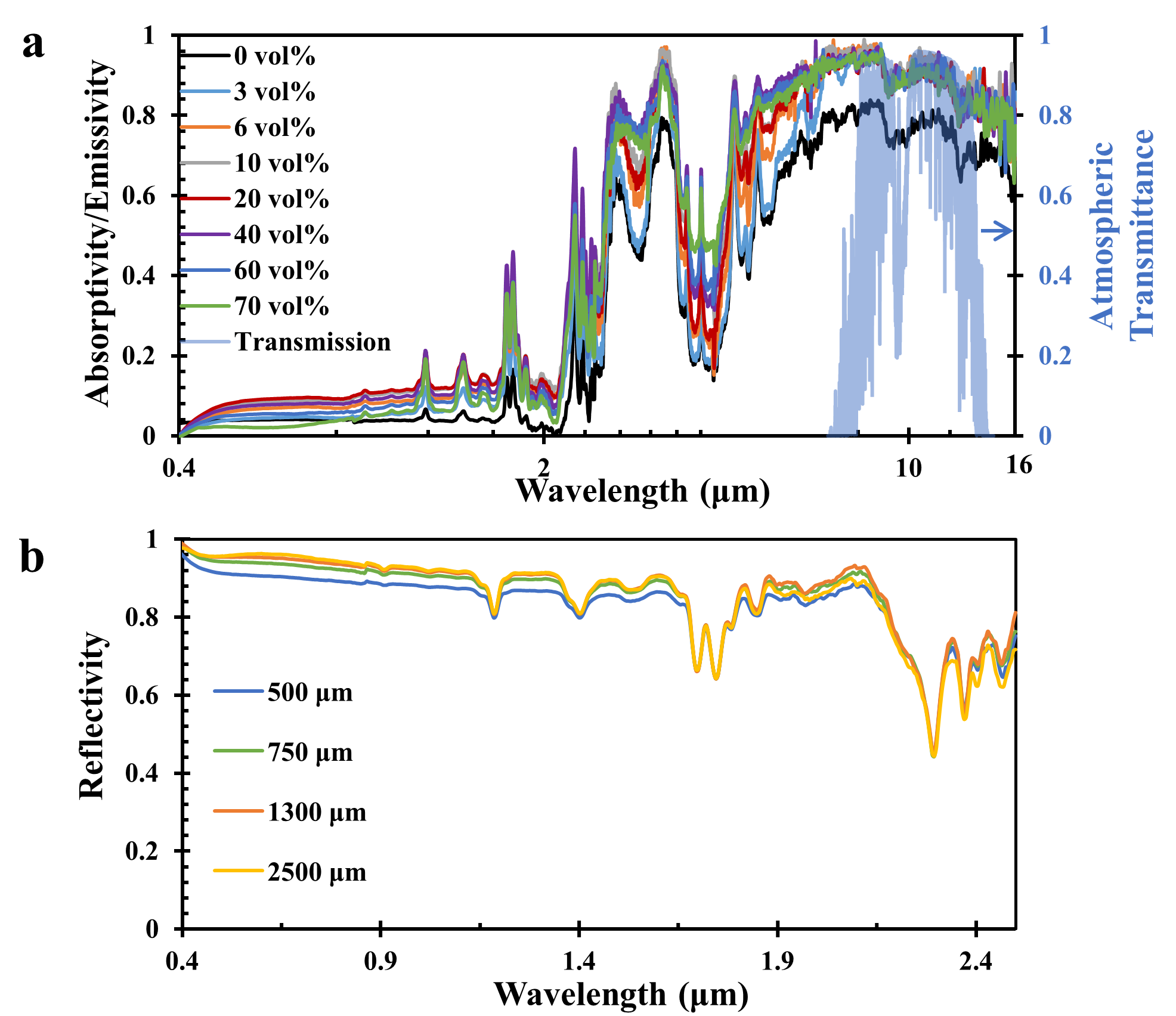


**Fig. S2. The optical properties of polymer coatings with varying ϕ from 0 to 70%.** (**a**) The emissivity of polymer coatings with varying ϕ from 0 to 70% in the wavelength range from 0.4 μm to 16 μm. The emissivity is calculated using E=1-R-T and measured values of diffuse reflectivity and transmissivity presented in Fig. 2. The as-called atmospheric window is added for reference; (**b**) The measured UV-VIS-NIR reflectivity of 70vol% polymer coating with different thicknesses from 500 μm to 2500 μm.


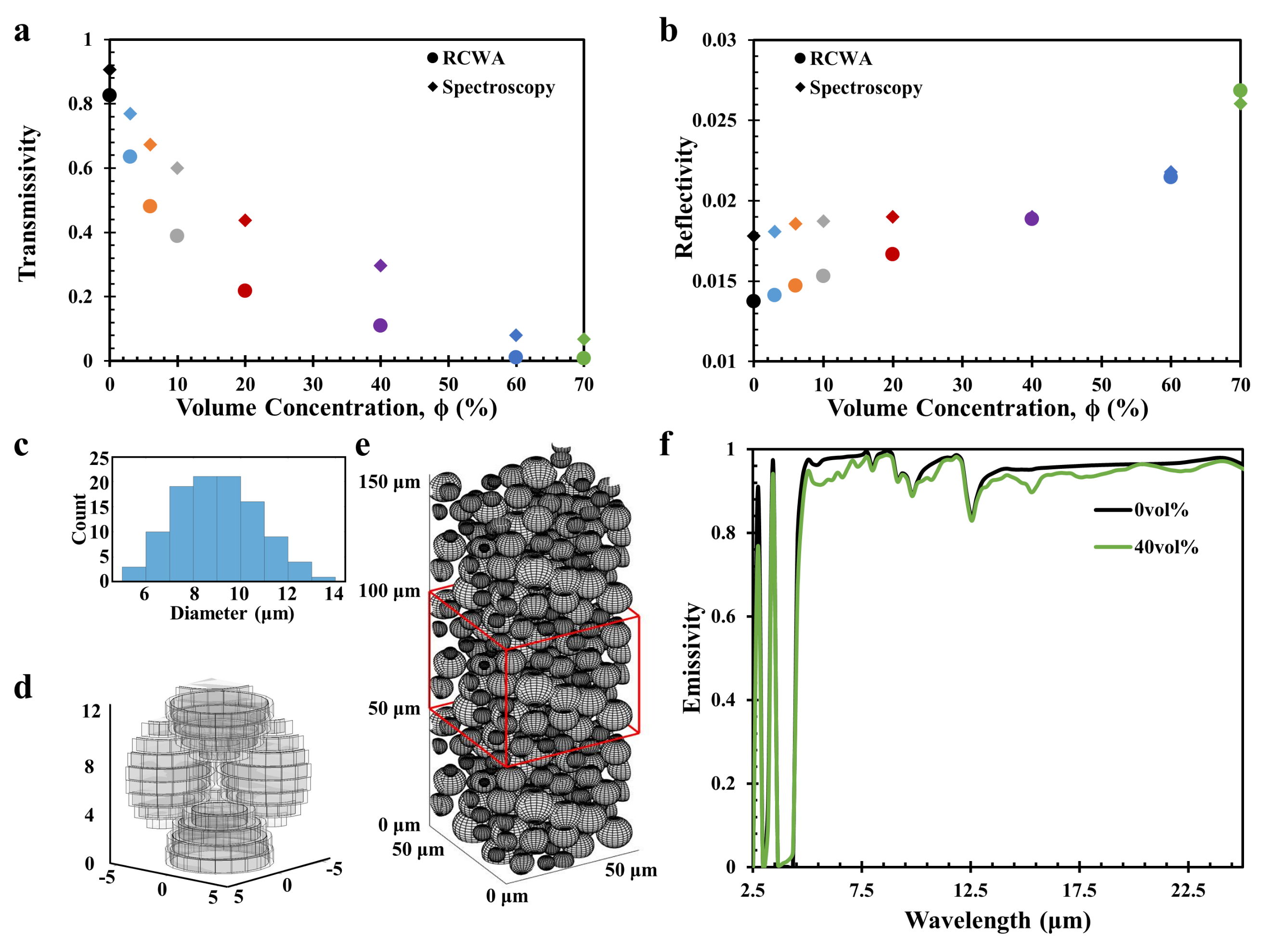


**Fig. S3. RCWA computation.** (**a**) Comparison between measured average transmissivity values and RCWA computed average transmissivity values using converted refractive index in the wavelength range between 0.4 μm and 2.5 μm; (**b**) Comparison between measured average reflectivity values and RCWA computed average reflectivity values using converted refractive index in the wavelength range between 0.4 μm and 2.5 μm, for polymer coatings with varying ϕ from 0 to 70%. For the RCWA computation, here we use a 2 μm × 2 μm × 600 μm unit cell for all ϕ; (**c**) Diameter distribution of 40 vol% polymer coating for a unit cell; (**d**) Representative stacked layered structure of glass bubbles for RCWA computation; (**e**) Demonstration of the stacking unit cells with the dimension of 50 μm^3^ to the simulated 600μm thickness. Here, we have three stacking unit cells in the schematic; (**f**) RCWA computation of the mid-IR emissivity of the pure PDMS (0 vol% polymer coating) and 40 vol% polymer coating.


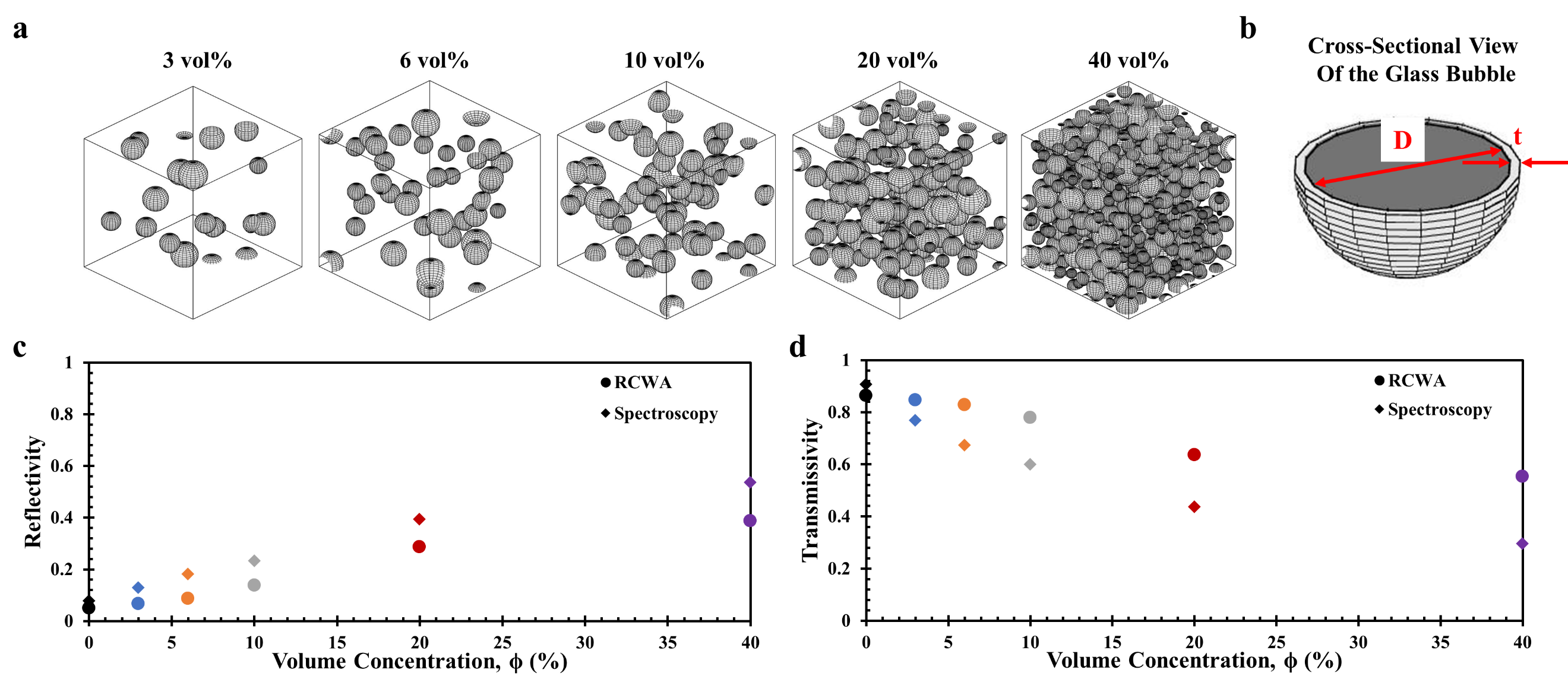


**Fig. S4. RCWA unit cell structures and results.** (**a**) Representative unit cells with dimension of 75^3^ µm^3^ for RCWA computation of polymer coatings with varying ϕ of glass bubbles inside PDMS. From left to right, ϕ is 3 vol%, 6 vol%, 10 vol%, 20 vol% and 40 vol%, respectively; (**b**) The three-dimensional view of single glass bubble used in the RCWA computation. The inner diameters (D) are set from 2 μm to 20 μm and the shell thicknesses (t) are correspondingly set to 0.05 times the inner diameters (D), leading to 0.1 μm to 1 μm; Comparison between (**c**) RCWA computed average reflectivity and measured average diffuse reflectivity, (**d**) RCWA computed average transmissivity and measured average diffuse transmissivity of polymer coatings with varying ϕ from 0 vol% to 40 vol% in the solar spectrum (0.4 μm to 2.5 μm). The unit cell thickness is set to 600 μm for all ϕ.


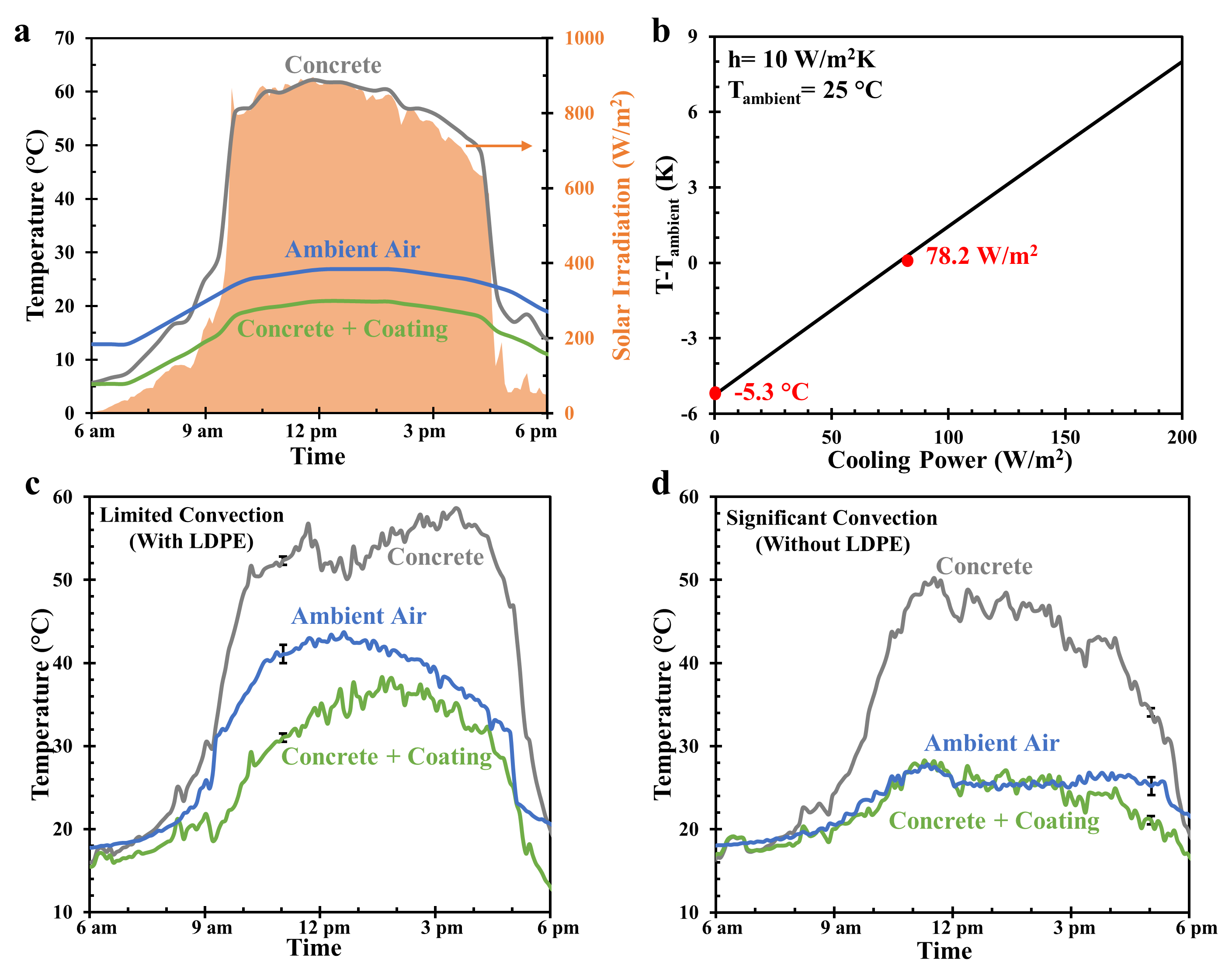


**Fig. S5. Thermal performance of the 70 vol% polymer coating.** (**a**) The predicted temperatures of the ambient air (blue), bare concrete block (grey) and concrete block with 70 vol% polymer coating on top (green) from 6 am to 6 pm, assuming a steady ambient environment, a representative daily weather data in summer, a constant conductive and convective heat transfer coefficient h=10 W/m^2^K and a peak solar irradiance of 875 W/m^2^ at 12 pm in Southern California. The solar irradiation shown in the plot and denoted by the secondary Y-axis was measured by a weather station near campus on June 28^th^, 2019 and used in the temperature predictions; (**b**) Temperature difference between surface of concrete block with 70 vol% polymer coating and ambient air as a function of cooling power. For this temperature prediction, the calculated emissivity values based on measured reflectivity and transmissivity data from spectroscopy are used. A constant conductive and convective heat transfer coefficient h=10 W/m^2^K and an ambient air temperature of 25 °C are assumed; (**c**) The optical image of the outdoor temperature measurement apparatus with a 25 μm-thick layer of LDPE film as the wind shield during the measurement.


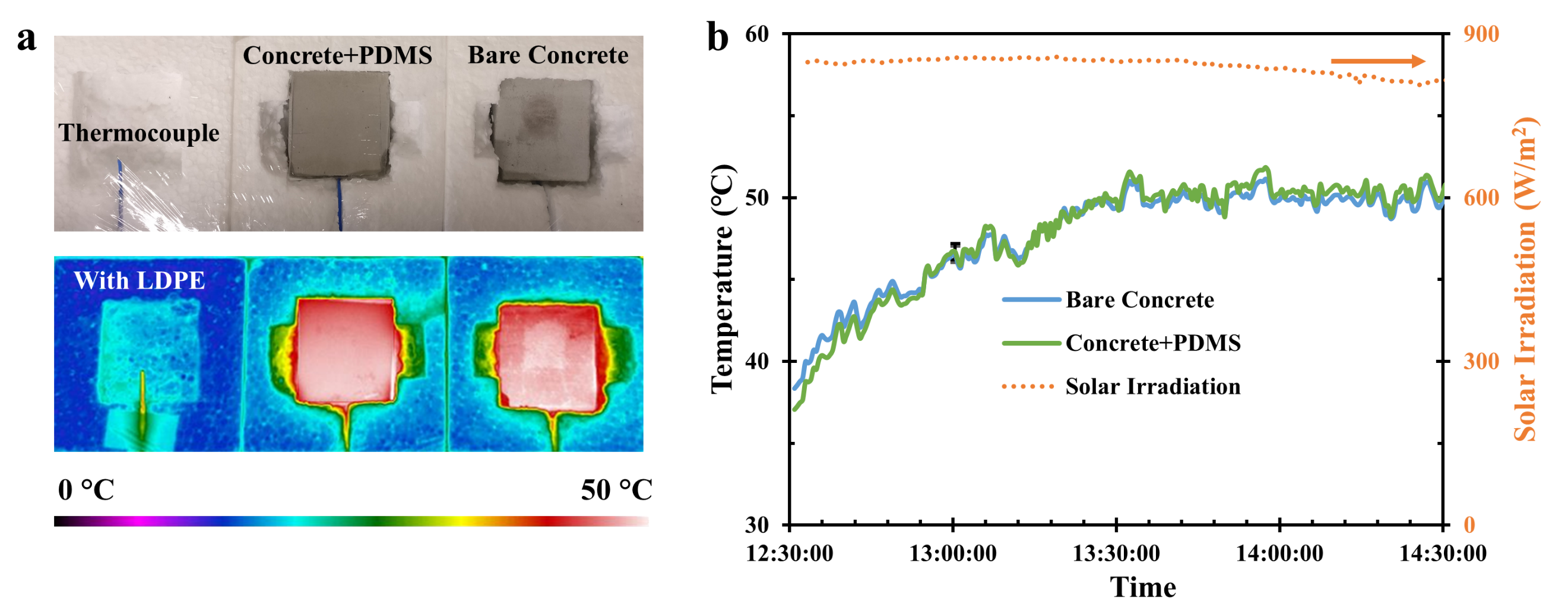


**Fig. S6. Temperature comparison between bare concrete and concrete with PDMS.** (**a**) The optical images and the corresponding IR image of the temperature measurement apparatus with a 25 μm-thick layer of LDPE film as the wind shield. In the optical and IR image, the left is the thermocouple used for measuring ambient air temperature. The middle is the concrete block of 2 inch × 2 inch × 0.5 inch with a same-area 2mm-thick PDMS on top and the right is the bare concrete block; (**b**) Temperature measurement data from 12:30 pm to 2:30 pm for bare concrete and concrete with PDMS, with a LDPE film covered on the top as the wind-shield during the measurement. The temperature data was obtained on Mar. 8^th^, 2020.


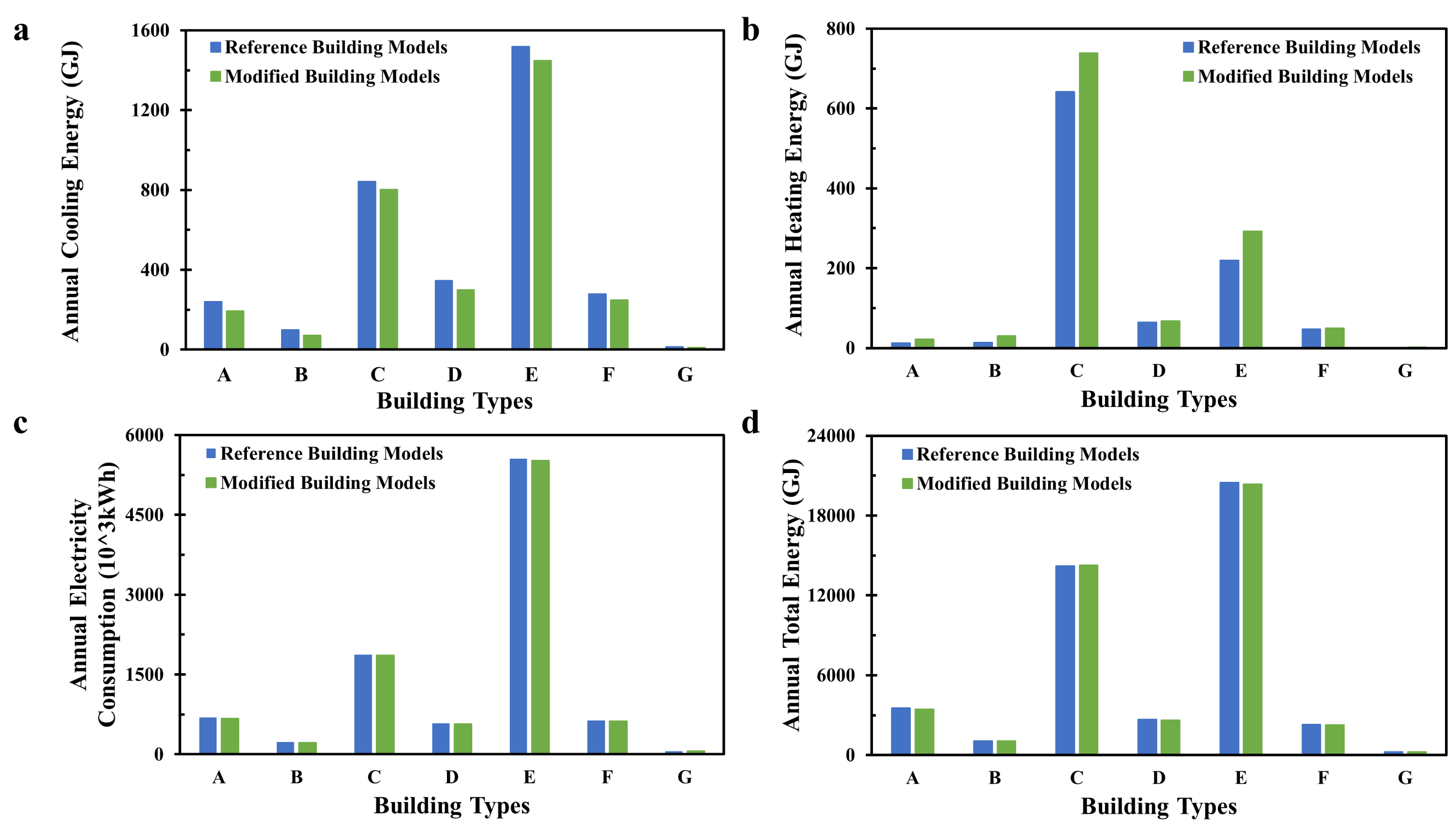


**Fig. S7. Annual building energy consumption change after we modify the DOE developed reference building types via adding a 2 mm-thick 70 vol% polymer coating for roofing and siding on the exterior surfaces.** Here A, B, C, D, E, F and G denote highrise apartment, midrise apartment, large hotel, small hotel, large office, medium office and small office, respectively. Annual (**a**) cooling energy, (**b**) heating energy, (**c**) electricity consumption and (**d**) total energy for reference building types and modified building types with 70 vol% polymer coating. Total energy includes electricity consumption, while electricity consumption includes cooling energy, lightning, equipment electricity consumption, fan energy and refrigeration, etc.


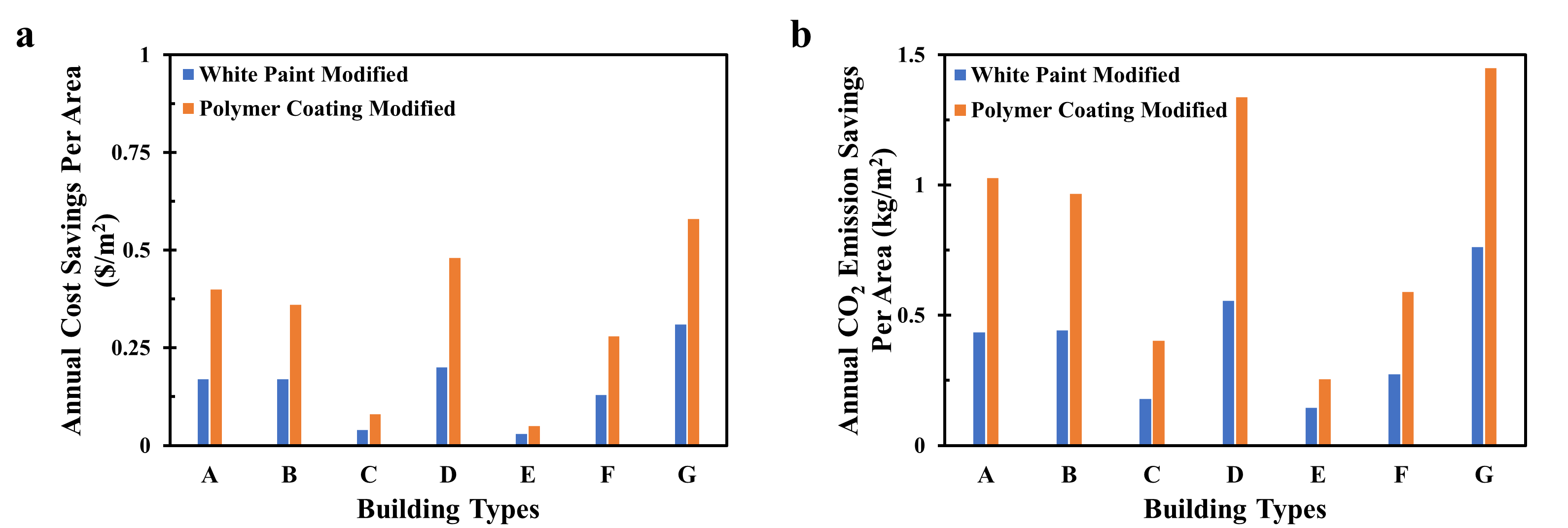


**Fig. S8. Annual cost savings and CO_2_ emission savings via commercial white paint modification and 70 vol% polymer coating modification.** Here A, B, C, D, E, F and G denote highrise apartment, midrise apartment, large hotel, small hotel, large office, medium office and small office, respectively. (**a**) Annual cost savings and (**b**) annual CO_2_ emission savings for white paint modified building types and polymer coating modified building types.


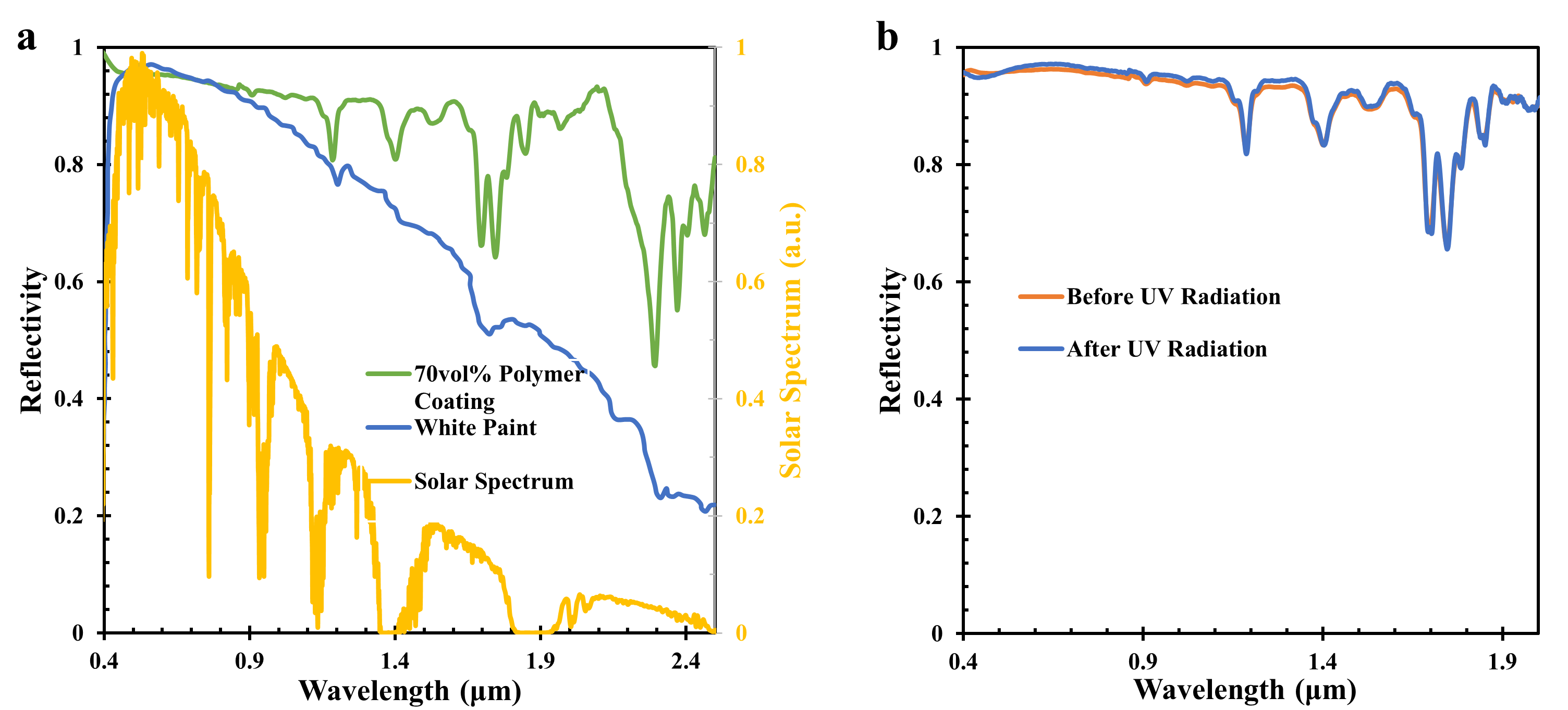


**Fig. S9. High NIR reflectivity and UV-damage-free property of 70 vol% polymer coating.** (**a**) UV-VIS-NIR reflectivity comparison between commercially available TiO_2_-based white paint and 70 vol% polymer coating; (**b**) UV-VIS-NIR reflectivity comparison for 70 vol% polymer coating before and after UV radiation.


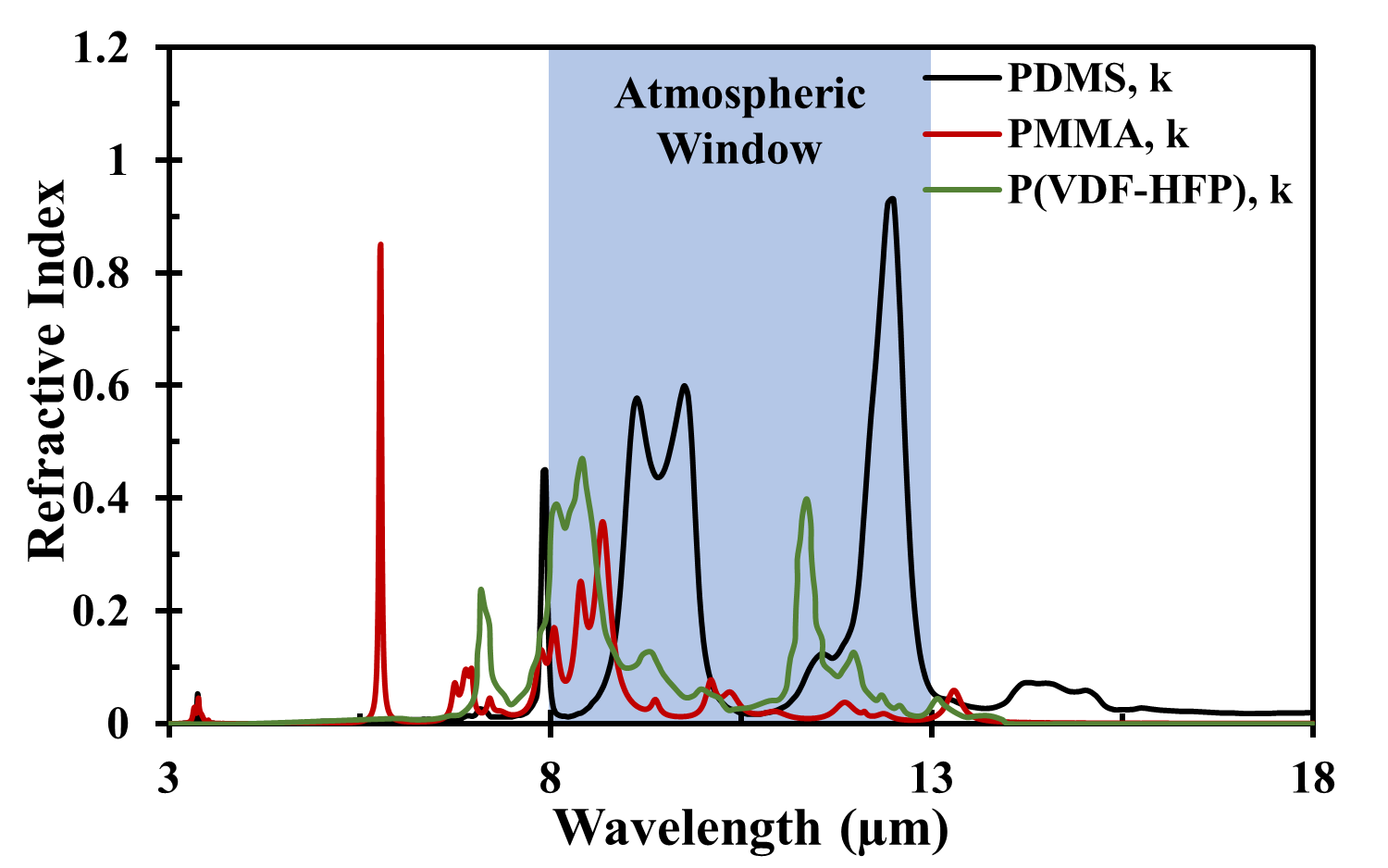


**Fig. S10.** **Extinction coefficients in refractive indices of PDMS, PMMA, P(VDF-HFP), showing their high emissivity in the mid-infrared wavelength region.** The peaks in k correspond to different vibrational modes of various molecular structures.

| Volume Concentration (%) | Measured Thickness (μm) | Theoretical Density (kg/m^3^) | Measured Density (kg/m^3^) | Areal Density (kg/m^2^) |
| --- | --- | --- | --- | --- |
| 0 | 330 | 965 | 927 | 0.306 |
| 3 | 330 | 950 | 905 | 0.299 |
| 6 | 340 | 935 | 892 | 0.303 |
| 10 | 300 | 915 | 881 | 0.264 |
| 20 | 260 | 864 | 855 | 0.222 |
| 40 | 380 | 763 | 845 | 0.321 |
| 60 | 740 | 662 | 810 | 0.599 |
| 70 | 1300 | 612 | 681 | 0.885 |

**Table S1. The density information of polymer coatings with varying ϕ from 0 to 70%.**

| Building Types | Exterior Surface Area (m^2^) | Total Volume (m^3^) | Exterior Area/Total Volume (m^-1^) | Number of Floors |
| --- | --- | --- | --- | --- |
| Highrise Apartment | 4536.49 | 21517.16 | 0.21 | 9 |
| Midrise Apartment | 2325.66 | 9553.61 | 0.24 | 4 |
| Large Hotel | 6538.43 | 35184.77 | 0.19 | 6 |
| Small Hotel | 2676.8 | 11447.42 | 0.23 | 4 |
| Large Office | 15747.01 | 126016.37 | 0.12 | 12 |
| Medium Office | 3638.4 | 13667.13 | 0.27 | 3 |
| Small Office | 880.27 | 1559.03 | 0.56 | 1 |

**Table S2. Dimensional information of seven different types from US Department of Energy (DOE) Commercial Reference Building Database and ASHRAE Standard 90.1 Prototype Building Database** (New Construction after 2004).

| Property | 70 vol% Polymer Coating | White Paint |
| --- | --- | --- |
| Thickness (mm) | 2 | 0.15 |
| Conductivity (W/m·K) | 0.3 | 0.57 |
| Density (kg/m^3^) | 681 | 1162 |
| Thermal Absorptance | 0.85 | 0.85 |
| Solar Absorptance | 0.16 | 0.45 |
| Visible Absorptance | 0.08 | 0.14 |

**Table S3. Detailed input material properties of the 70 vol% polymer coating used in the building energy consumption analysis.**

**References**

1. Howell, John R., M. Pinar Menguc, and R. S. *Thermal radiation heat transfer*. *CRC Press* (2015).

2. Lalanne, P. & Jurek, M. P. Computation of the near-field pattern with the coupled-wave method for transverse magnetic polarization. *J. Mod. Opt.* **45**, 1357–1374 (1998).

3. Querry, M. Optical Constants of Minerals and Other Materials from the Millimeter to the Ultraviolet. *Contract. Rep.* (1987).

4. Palik, E. D. *Handbook of optical constants of solids*. *Academic Press* (1998). doi:10.1016/C2009-0-20920-2

5. Howell, J. R., Mengüç, M. P. & Siegel, R. *Thermal radiation heat transfer, sixth edition*. *Thermal Radiation Heat Transfer, Sixth Edition* (2015).

6. Raman, A. P., Anoma, M. A., Zhu, L., Rephaeli, E. & Fan, S. Passive radiative cooling below ambient air temperature under direct sunlight. *Nature* **515**, 540–544 (2014).

7. Hossain, M. M. & Gu, M. Radiative cooling: Principles, progress, and potentials. *Adv. Sci.* **3**, 1–10 (2016).

8. Peoples, J. *et al.* A strategy of hierarchical particle sizes in nanoparticle composite for enhancing solar reflection. *Int. J. Heat Mass Transf.* **131**, 487–494 (2019).

9. Weather Spark Weather Data. Available at: https://weatherspark.com/. (Accessed: 6th January 2020)

10. Team, E. W. ESRL Global Monitoring Division-Global Radiation Group. (2005).

11. Deru, M. *et al.* *US Department of Energy commercial reference building models of the national building stock*. (National Renewable Energy Laboratory, 2011).

12. Energy standard for buildings except low-rise residential buildings. *ASHRAE Standard* (2004).

13. Mandal, J. *et al.* Hierarchically porous polymer coatings for highly efficient passive daytime radiative cooling. *Science.* **362**, 315–319 (2018).

14. Levinson, R., Akbari, H., Konopacki, S. & Bretz, S. Inclusion of cool roofs in nonresidential Title 24 prescriptive requirements. *Energy Policy* **33**, 151–170 (2005).

15. Zhai, Y. *et al.* Scalable-manufactured randomized glass-polymer hybrid metamaterial for daytime radiative cooling. *Science.* **355**, 1062–1066 (2017).

16. Orel, B., Gunde, M. K. & Krainer, A. Radiative cooling efficiency of white pigmented paints. *Sol. Energy* **50**, 477–482 (1993).
